# Comments on “Recent Developments in Low-Level Lead Exposure and Intellectual Impairment in Children”

**DOI:** 10.1289/ehp.113-1253736

**Published:** 2005-01

**Authors:** Todd A. Jusko, Richard L. Canfield, Charles R. Henderson, Bruce P. Lanphear

**Affiliations:** Department of Epidemiology, University of Washington, Seattle, Washington, E-mail: jusko@u.washington.edu; Division of Nutritional Sciences, Cornell University, Ithaca, New York; Department of Human Development Cornell University, Ithaca, New York; Cincinnati Children’s Hospital, Medical Center, Cincinnati, Ohio

We commend Koller et al. (2004) for their thoughtful and detailed review of recent research on childhood lead exposure and intellectual development, and we take this opportunity to clarify and respond to several of their questions regarding our study of children with blood lead concentrations <10 μg/dL) (Canfield et al. 2003).

The children in our cohort were recruited between 24 and 30 months of age, and all had participated in a prior randomized dust control trial (Lanphear et al. 1999). In that trial, dust and blood lead concentrations were assessed at 6, 12, 18, and 24 months of age as part of an evaluation of whether dust control measures reduced children’s blood lead concentrations. Koller et al. (2004) raised several questions related to whether children’s participation in the prior study affected the results we reported (Canfield et al. 2003). Specifically, their concerns related to confounding, where an imbalance in the distribution of intervention/control participants across levels of blood lead and IQ could bias the association between blood lead concentrations and IQ.

Our statistical model was developed a priori and included covariates that were established predictors of children’s intelligence (Canfield et al. 2003). Because home visitation by a dust control team (intervention) seemed unlikely to increase children’s IQ and because children who participated in the intervention actually had slightly lower IQ scores at 3 and 5 years of age compared with controls, intervention status was not considered a plausible confounder. To demonstrate that participation in the dust control trial did not introduce any bias of consequence and to illustrate our basis for excluding intervention status from our published models, in this letter we summarize results for a semiparametric spline model, which is identical to the one we reported previously (Canfield et al. 2003) except for the inclusion of intervention status as a potential confounding factor. The estimated decline in IQ as blood lead concentration increases from 1 to 10 μg/dL is 6.8 points when controlling for intervention status. This estimate is not meaningfully different from the 7.4-point decline we reported previously (Canfield et al. 2003). Furthermore, the shape of the dose–response function is preserved, with a steeper slope at lower blood lead concentrations. Estimates of the predicted decline in IQ from parametric models with linear and quadratic terms for blood lead also differ by < 10% from the reported results (Canfield et al. 2003) when intervention status is included in the model.

Additionally, Koller et al. (2004) suggested that the Stanford-Binet IV Test of Intelligence (SBIV) may not have provided the most accurate estimate of IQ for our cohort because of the relative weighting of verbal and nonverbal skills that are assessed and because of problems with the standard method of dealing with zero-scored subtests. Koller et al. (2004) suggested that the Wechsler Primary and Preschool Scales of Intelligence (WPPSI) would have yielded a more reliable and valid measure of intelligence. We first note that despite many attractive features of the WPPSI (and especially of the WPPSI-Revised, which we considered using), the SBIV has features that we believe made it a superior test for our particular cohort. Most importantly, the SBIV can be administered to 2-year-olds, whereas the youngest age for the WPPSI-R is 3 years. Because our sample was predominantly composed of families with lower parental education and income, we preferred the test with the lower floor.

With respect to how zero-scored subtests are handled, we indeed followed the standard scoring procedure for the SBIV, which states that a zero score “should not be included in the determination of the related Area Score or of the Composite Score” (Delaney and Hopkins 1987). Because this scoring method was used in the standardization of the instrument, a different approach would yield scores with unknown psycho-metric properties and thereby compromise interpretation of the results.

Nevertheless, any particular scoring method has its weaknesses, and we agree that it would be useful to know whether our results change markedly by incorporating information about zero scores. We therefore added as a time-varying covariate in our mixed models the number of subtests on which each child scored zero. In the semi-parametric spline model, the estimated decline in IQ as blood lead concentration increased from 1 to 10 μg/dL was 6.3 points. Estimates from parametric models with linear and quadratic terms for blood lead differed by < 5% from the results we reported previously (Canfield et al. 2003). Thus, the incorporation of information about zero-scored subtests did not change our results markedly.

Potential sources of confounding and misclassification need to be carefully considered in the design and analysis phase of any study, observational or otherwise, and in the interpretation of results. The detailed attention given to these issues by Koller et al. (2004) has allowed us the opportunity to provide additional information about our methods and results and thereby address these methodologic issues.
